# Housing characteristics by living arrangement of older adults with cognitive impairment in the United States

**DOI:** 10.1002/alz.089788

**Published:** 2025-01-09

**Authors:** Safiyyah Okoye, Laura N. Gitlin

**Affiliations:** ^1^ Drexel University, Philadelphia, PA USA

## Abstract

**Background:**

The home environment is where daily activities are performed and older adults with and without cognitive impairment spend most of their time. While home safety is a priority for healthcare providers, there is limited understanding of housing characteristics of older adults with cognitive impairment in the United States and whether they differ for those who live alone or with others.

**Methods:**

We draw on data collected from N = 968 community‐living older adults with cognitive impairment who responded to the 2022 National Health and Aging Trends Study, a population‐based national survey that comprehensively assesses characteristics of older adults (≥65 years) and their households in a two‐hour in‐home interview. Housing characteristics reported by self/proxy response were: building type, own or rent, home modifications, and whether living in subsidized housing. Housing characteristics directly observed by interviewers were: home disorder (e.g., flooring problems, pests); home disrepair (e.g., missing siding, broken steps); and street disorder (e.g., litter, graffiti, vacant houses). Cognitive status was measured by a composite measure.

**Results:**

We found that 26.0% (n = 261) of our sample lived alone, 46.4% (n = 373) lived with a spouse, and 27.6% (n = 334) lived with others (non‐spouse). The prevalence of some housing characteristics differed between the three groups. For example, renting was most prevalent among those who lived with others (non‐spouses); 67.1% vs 48.4% if lived alone and 20.9% if lived with a spouse. Living in an apartment or mobile home (vs house) was more prevalent for those who lived alone (27.1% vs. 15.4% if lived with others (non‐spouse) and 13.5% if lived with a spouse). Other characteristics were similar across groups: approximately 45% (n = 437) of the overall sample lived in a home with internal disorder, 24% (n = 187) in a home with external disrepair, and 20% (n = 202) on a street where disorder was noted (rates did not statistically significantly differ between groups).

**Conclusions:**

Although the distribution of some housing characteristics differed by living arrangement, 25‐50% in all groups were observed to have housing challenges such as disrepair and disorder. As housing conditions can have significant health consequences and prompt relocation, attention to housing should be part of routine dementia care.

